# Supplementary material for: Disseminated intravascular coagulation, associated factors and clinical outcomes among critically Ill septic adults admitted to a tertiary hospital in Ethiopia: A prospective longitudinal study
Source: PLoS One. 2025 Aug 22;20(8):e0330842. doi: 10.1371/journal.pone.0330842 (PMC12373190; doi:10.1371/journal.pone.0330842)
Supplement: S1 Table — (DOCX) [file pone.0330842.s001.docx]

**Table** **1**: Diagnostic criteria of DIC and their scoring

| ISTH JAAM SIC |
| --- |
| Item Score Range Range Range |
| Platelet count 3 - < 80 - |
| ≧ 50% decrease within 24h |
| 2 <50 - <100 |
| 1 > 50, <100 120>, 80 < > 100, < 150 |
| > 30% decrease within 24hr |
| FDP (D-dimer) 3 Strong increase > 25µg/mL (use convert chart) - |
| 2 Moderate increase - - |
| 1 - > 10, < 25µg/mL - |
| PT (PT ratio) 2 > 6 sec - >1.4 |
| 1 > 3 sec, < 6 sec > 1.2 (PT ratio) >1.2, 1.4 |
| (PT-INR) |
| FBG (g/mL) 1 <100 - - |
| SIRS score 1 - >3 |
| SOFA score 2 - - > 2 |
| 1 - - 1 |
| Total score for > 5 > 4 > 4 |
| DIC or SIC |

***ISTH*** International Society on Thrombosis and Haemostasis, ***DIC*** disseminated intravascular coagulation ***JAAM*** Japanese Society on Acute Medicine, ***SIC*** Sepsis-Induced Coagulopathy, ***SIRS*** Systemic Inflammatory Response Syndrome, ***SOFA*** sequential organ failure assessment Total SOFA score is the sum of 4 items (respiratory SOFA, cardiovascular SOFA, hepatic SOFA, and renal SOFA)
