# Supplementary material for: Disseminated intravascular coagulation, associated factors and clinical outcomes among critically Ill septic adults admitted to a tertiary hospital in Ethiopia: A prospective longitudinal study
Source: PLoS One. 2025 Aug 22;20(8):e0330842. doi: 10.1371/journal.pone.0330842 (PMC12373190; doi:10.1371/journal.pone.0330842)
Supplement: Attachment — Submitted filename: Response to Reviewers.docx [file pone.0330842.s004.docx]

**Response to reviewers**

Dear Editor and reviewers,

We are grateful for the valuable and constructive comments provided. Your critical evaluation of the manuscript will definitely improve the work. We made a point-by-point response to your comments and suggestions as follows and changes were made in the manuscript using track change:

**Reviewer #1:**

1. **How were the patients defined/diagnosed as "septic"? I suggest to the authors the inclusion of the reference.**

**Response**: Thank you for the critical inquiry regarding the definition used for sepsis diagnosis. As it is indicated in the limitation of the study, Sepsis-3 definition which employs SOFA score for sepsis screening was not used in this study. The practicality of using SOFA score is highly limited particularly in resource constrained settings like ours. Additionally, the development of the SOFA score did not take into account data of low resource countries. The surviving sepsis campaign (SSC) 2021 guideline recommends use of “a performance improvement program for sepsis” which included sepsis screening for acutely ill. However, there is no single screening tool that wins universal assent due to the heterogeneity of the disease and variability of settings across the world.

Recognizing these challenges, the SSC guidelines encourage hospitals to adopt ***the most accurate, feasible, and sustainable approach to sepsis screening***. Although qSOFA was proposed as a simplified screening tool, previous studies indicated that it is not suitable for ICU settings. Consequently, many ICUs continue to rely on the SIRS score and physicians’ clinical judgment based on readily available parameters. Likewise, in our study, sepsis diagnoses were determined by attending physicians, taking into account clinical, laboratory, and radiological findings. Clinical investigations included pulse rate, respiratory rate, temperature, oxygen saturation, and Glasgow Coma Scale, while the laboratory analyses included culture, CBC, procalcitonin, C-reactive protein, and organ function tests.

A sub-section in the method part is added to describe the **sepsis diagnosis** used in this study. A reference (1) has also been cited based on the reviewer request. Kindly refer to this reference on the last section **(section 9: Screening),** **page 27, recommendation 1.**

2. **Also, how were the patients diagnosed as "serious liver disorders"?**

**Response**: Thank you for the concern raised regarding serious liver disorder diagnosis. We hope that this question arises from the statement in the exclusion criteria. We are sorry for the mistake we made in describing serious liver disorder as one of the exclusion criteria. We had to omit the phrase from the exclusion criteria. During protocol development, we planned to exclude patients with serious liver disorder to avoid confounding effect on the coagulopathy. However, later during research, we decided that excluding anticoagulant use and known coagulopathy suffice. We now omitted serious liver disorder from the exclusion criteria.

3. **How were the patients diagnosed as kidney failure, brain failure, lung failure and liver failure?**

**Response**: Thank you for the valuable questions raised regarding diagnosis of organ failures. The following criteria were used in the study area’s ICUs to diagnose organ failure:

**Liver failure:** was diagnosed considering significantly deranged liver functional tests including albumin, bilirubin, and INR.

**Kidney failure:** was diagnosed when the eGFR was less than 15ml/min/1.73m^2^ and when there was a need for renal replacement therapy (RRT).

**Respiratory failure:** was diagnosed when lungs couldn’t exchange O_2_ and CO_2_, leading to abnormal gas exchange. In addition to the respiratory rate >35 or <8/min, the pulse oximetry reading with a saturation less than 91% was considered as a surrogate for PaO_2_< 60mmHg.

**Brain death/failure:** was diagnosed when there was coma, absence of brain stem reflexes and apnea (lack of spontaneous breathing).

1.	Surviving Sepsis Campaign. Early Identification of Sepsis on the Hospital Floors: Insights for Implementation of the Hour-1 Bundle [Internet]. 2019 [cited 2024 Dec 23]. Available from: https://sccm.org/survivingsepsiscampaign/hour-1-bundle-implementation-guide

**Reviewer #2:**

**General Comments:**
This is a prospective cohort study based on ICU patients from a local hospital, enrolling a total of 148 patients diagnosed with sepsis. The manuscript is generally well-structured, employs appropriate statistical methods, and contributes to the understanding of DIC development among septic patients within the context of the local disease spectrum. However, there are concerns regarding the rigor of the discussion, the originality of the conclusions, and the presentation quality of several figures. Overall, I believe that the manuscript does not yet meet the publication standards of this journal. Nevertheless, I am confident that, with careful revision, particularly in improving the clarity of visualizations and the scientific robustness of the conclusions, the manuscript could be significantly enhanced.

**Specific Comments:**

1. **Figure Quality and Presentation**: The flowchart in Figure 1 could be visually improved. Many online tools are available to create streamlined, reader-friendly diagrams that would enhance the visual clarity of your results. Similar issues are noted in Figure 2 and Figure 4. In Figure 2, the bar plot would better reflect the differences in patient numbers if the bars were left-aligned rather than center-aligned. In Figure 4, the AUROC plot lacks a legend. While it can be inferred that the red line may represent a random classifier, this should be clearly labeled within the figure, and AUROC values should be explicitly reported.

**Response**: Thank you so much for the valuable comments provided on the clarity of the figures. We modified the figures (Fig 1, 2, and 4) to improve the clarity.

2. **Discussion of AST and DIC Association**: The discussion on the association between AST levels and the occurrence of DIC in ICU septic patients is less rigorous compared to the discussion of other factors. First, the authors should elaborate on the changes in AST levels that may result from sepsis, similar to their explanation of sepsis-induced thrombocytopenia and prolonged PT. Second, the current argument linking AST to DIC mainly relies on the intermediary presence of liver disease, without direct evidence connecting AST elevation to DIC. Given that AST elevation can be influenced by various non-hepatic conditions, and that liver disease may lead to a wide range of complications beyond coagulopathy, the causal inference here appears somewhat tenuous. A more comprehensive literature review and clearer mechanistic explanation are warranted.

**Response**: Thank you for the insightful comments. We addressed the comments by describing the effect of sepsis on AST and the possible mechanisms underlying elevated AST in DIC beyond the liver disease. We added statements from line 304 to 311, and from line 317 to 320.

3. **Clarification on Sepsis Definition**:
   A 2016 article published in Critical Care entitled "Revision of the Japanese Association for Acute Medicine (JAAM) disseminated intravascular coagulation (DIC) diagnostic criteria using antithrombin activity" discussed an update of the JAAM criteria to align with the Sepsis-3 definition. While the JAAM scoring system is a key variable in this study, the supplementary material indicates that the Sepsis-3 definition was not adopted. The authors should clarify the rationale behind not using the Sepsis-3 criteria—whether due to data limitations, clinical considerations, or other constraints.

**Response**: Thank you for the important comment on the updated JAAM scoring system. As it has been indicated in the limitation of the study, the sepsis-3 definition was not used in this study due to lack of some SOFA components. Data on antithrombin activity was also lacking to use the updated JAAM score.

4. **Interpretation of JAAM Score Cut-off:**
   The manuscript suggests that a JAAM score ≥7 can predict mortality in ICU septic patients with DIC. However, given that the JAAM scoring range is 0–8, and that a score ≥4 already meets the diagnostic threshold for DIC, the clinical utility of using such a high cutoff (≥7) as a prognostic marker may be limited. A score this high suggests an already advanced coagulopathy and poor prognosis, potentially reducing the incremental predictive value of the finding. Moreover, previous studies have already demonstrated the prognostic relevance of the JAAM score in severe sepsis. Thus, the novelty of this conclusion appears limited and should be further discussed.

**Response**: Thank you for the insightful comments on the predictive role of JAAM score. After this comment, we chose to reduce the cut-off value to ≥5 with a sensitivity of 70.5% and specificity of 71.4% (line #258, 259, 296, 297, and table 4). Though the prognostic performance of JAAM score was reported in previous studies, its role in predicting **in-ICU** mortality is scarcely reported. Additionally, validating its relevance across various contexts could offer meaningful contributions to the existing body of literature.

5. **Ambiguity in the Conclusion Statement:**
   The final sentence of the conclusion is somewhat ambiguous. It is unclear whether the authors propose that the JAAM score can serve as a prognostic marker for all ICU patients, or specifically for septic ICU patients with DIC. This should be clearly stated in both the discussion and conclusion sections to avoid misinterpretation.

**Response**: Thank you for the valuable comment. The result, discussion, and conclusion regarding the predictive role of JAAM score is now corrected describing its usefulness only among sepsis patients with DIC (Line #258, 261, 263, 295 and 366).
